# Derivation, validation and assessment of a novel nomogram-based risk assessment model for venous thromboembolism in hospitalized patients with lung cancer: A retrospective case control study

**DOI:** 10.3389/fonc.2022.988287

**Published:** 2022-10-10

**Authors:** Huimin Li, Yu Tian, Haiwen Niu, Lili He, Guolei Cao, Changxi Zhang, Kaiseer Kaiweisierkezi, Qin Luo

**Affiliations:** Department of Respiratory and Neurology, The Affiliated Tumor Hospital of Xinjiang Medical University, Urumqi, China

**Keywords:** lung cancer, venous thromboembolism, risk assessment, nomogram model, thromboprophylaxis

## Abstract

**Purpose:**

This study aimed to develop and validate a specific risk-stratification nomogram model for the prediction of venous thromboembolism(VTE) in hospitalized patients with lung cancer using readily obtainable demographic, clinical and therapeutic characteristics, thus guiding the individualized decision-making on thromboprophylaxis on the basis of VTE risk levels.

**Methods:**

We performed a retrospective case–control study among newly diagnosed lung cancer patients hospitalized between January 2016 and December 2021. Included in the cohort were 234 patients who developed PTE and 936 non-VTE patients. The patients were randomly divided into the derivation group (70%, 165 VTE patients and 654 non-VTE patients) and the validation group (30%, 69 VTE patients and 282 non-VTE patients). Cut off values were established using a Youden´s Index. Univariate and multivariate regression analyses were used to determine independent risk factors associated with VTE. Variance Inflation Factor(VIF) was used for collinearity diagnosis of the covariates in the model. The model was validated by the consistency index (C-index), receiver operating characteristic curves(ROC) and the calibration plot with the Hosmer-Lemeshow goodness-of-fit test. The clinical utility of the model was assessed through decision curve analysis(DCA). Further, the comparison of nomogram model with current models(Khorana, Caprini, Padua and COMPASS-CAT) was performed by comparing ROC curves using the DeLong’s test.

**Results:**

The predictive nomogram modle comprised eleven variables: overweight(24-28) defined by body mass index (BMI): [odds ratio (OR): 1.90, 95% confidence interval (CI): 1.19-3.07], adenocarcinoma(OR:3.00, 95% CI: 1.88-4.87), stageIII-IV(OR:2.75, 95%CI: 1.58-4.96), Central venous catheters(CVCs) (OR:4.64, 95%CI: 2.86-7.62), D-dimer levels≥2.06mg/L(OR:5.58, 95%CI:3.54-8.94), PT levels≥11.45sec(OR:2.15, 95% CI:1.32-3.54), Fbg levels≥3.33 g/L(OR:1.76, 95%CI:1.12-2.78), TG levels≥1.37mmol/L (OR:1.88, 95%CI:1.19-2.99), ROS1 rearrangement(OR:2.87, 95%CI:1.74-4.75), chemotherapy history(OR:1.66, 95%CI:1.01-2.70) and radiotherapy history(OR:1.96, 95%CI:1.17-3.29). Collinearity analysis with demonstrated no collinearity among the variables. The resulting model showed good predictive performance in the derivation group (AUC 0.865, 95% CI: 0.832-0.897) and in the validation group(AUC 0.904,95%CI:0.869-0.939). The calibration curve and DCA showed that the risk-stratification nomogram had good consistency and clinical utility. Futher, the area under the ROC curve for the specific VTE risk-stratification nomogram model (0.904; 95% CI:0.869-0.939) was significantly higher than those of the KRS, Caprini, Padua and COMPASS-CAT models(Z=12.087, 11.851, 9.442, 5.340, all *P*<0.001, respectively).

**Conclusion:**

A high-performance nomogram model incorporated available clinical parameters, genetic and therapeutic factors was established, which can accurately predict the risk of VTE in hospitalized patients with lung cancer and to guide individualized decision-making on thromboprophylaxis. Notably, the novel nomogram model was significantly more effective than the existing well-accepted models in routine clinical practice in stratifying the risk of VTE in those patients. Future community-based prospective studies and studies from multiple clinical centers are required for external validation.

## Introduction

Venous thromboembolism (VTE), which manifests as deep vein thrombosis (DVT) and pulmonary thromboembolism (PE), is a major global burden of disease (1). DVT mostly affects the deep veins of the lower limbs. After the thrombi dislodge from clots in the deep veins of the lower limbs falls off, it can drift along with the blood flow and block the pulmonary arteries and its branches, resulting in PE. Hence, DVT and PE, collectively referred to as VTE, are the manifestations of the same disease at different stages. Of note, it has been established that there is a strong association between cancer and VTE events (2). On the one hand, patients with malignancy are at a high risk of VTE, account for approximately 20% of all patients complicated with vein thrombosis, and have a 4 to 7 fold increased risk of developing VTE compared to the general population (3). While on the other hand, cancer-associated thrombosis (CAT) is commonly associated with higher morbidity and mortality, increased hospital stay, reduced quality of life and higher medical costs (4, 5). Indeed, VTE is responsible for 9% of death in cancer patients, making it the second leading cause of death in cancer patients (6). As a result, VTE events continue to be common and potentially fatal complication in cancer inpatients.

However, the incidence of VTE might be underestimated due to the low rate of clinical detection, as well as the high rates of misdiagnosis and missed diagnosis (7). Several studies have reported that patients with lung cancer (LC) have a relatively higher of VTE development than patients with other solid tumors (8, 9). Furthermore, it has been recently recognized that VTE is surprisingly common in newly-diagnosed patients with LC and linked with poor prognosis (10). More importantly, considerable morbidity of long-term complications results from VTE, such as post-thrombotic syndrome and chronic thromboembolic pulmonary hypertension, which not only affect the treatment of patients with primary diseases but also reduce the patients’ quality of life (11, 12). Given the diminishing evidence regarding the benefits of VTE thromboprophylaxis in low risk situations, overprophylaxis is clearly undesirable, and could result in an inherent risk of bleeding which may offset its clinical benefits (13). Consequently, early detection of high-risk factors for lung cancer combined with VTE should be paid for particular attention. There is an urgent need for useful clinical tools to accurately predict the risk of VTE in hospitalized patients with LC and to guide individualized decision-making on thromboprophylaxis.

Presently, nomogram-based prediction model has been widely used as a user-friendly screening tool for the diagnosis and prognosis of diseases (14). Nomogram is a visual display of complex mathematical formulas, which integrates multiple prediction variables and then uses the line with scale according to a certain proportion, so that the probability of occurrence of predicted events can be simply determined. Currently, comprehensive treatments such as surgery, chemotherapy, radiotherapy and more recently immunotherapy have been recognized as additional risk factors for VTE in patients with lung cancer (15–17). In particular, the current analysis demonstrates that nomogram has good risk-prediction ability for VTE in postoperative lung cancer patients (18). However, studies on the use of a nomogram model for predicting the risk of CAT are limited, especially for patients with LC receiving first-line systemic therapy (19–21). Meanwhile, there is less risk assessment models(RAMs) to evaluate the risk of VTE exclusively for hospitalized cancer patients. Therefore, more attention should be paid to the construction of specific VTE risk assessment model to guide prophylaxis decisions for hospitalized LC patients.

Considering the differences in the patient population characteristics and treatment modalities, it is necessary to develop an accurate, objective, and practical tool to predicting VTE in lung cancer patients using available clinical parameters, which would be helpful in guiding clinical decision-making on prophylaxis. Therefore, the aim of this study was to develop and validate a specific risk-stratification nomogram model for the prediction of VTE in lung cancer patients to provide a theoretical basis for the individualized treatment on the basis of VTE risk levels, thus guiding the implementation of clinical prevention and treatment.

## Materials and methods

### Study design

**Figure 1:**
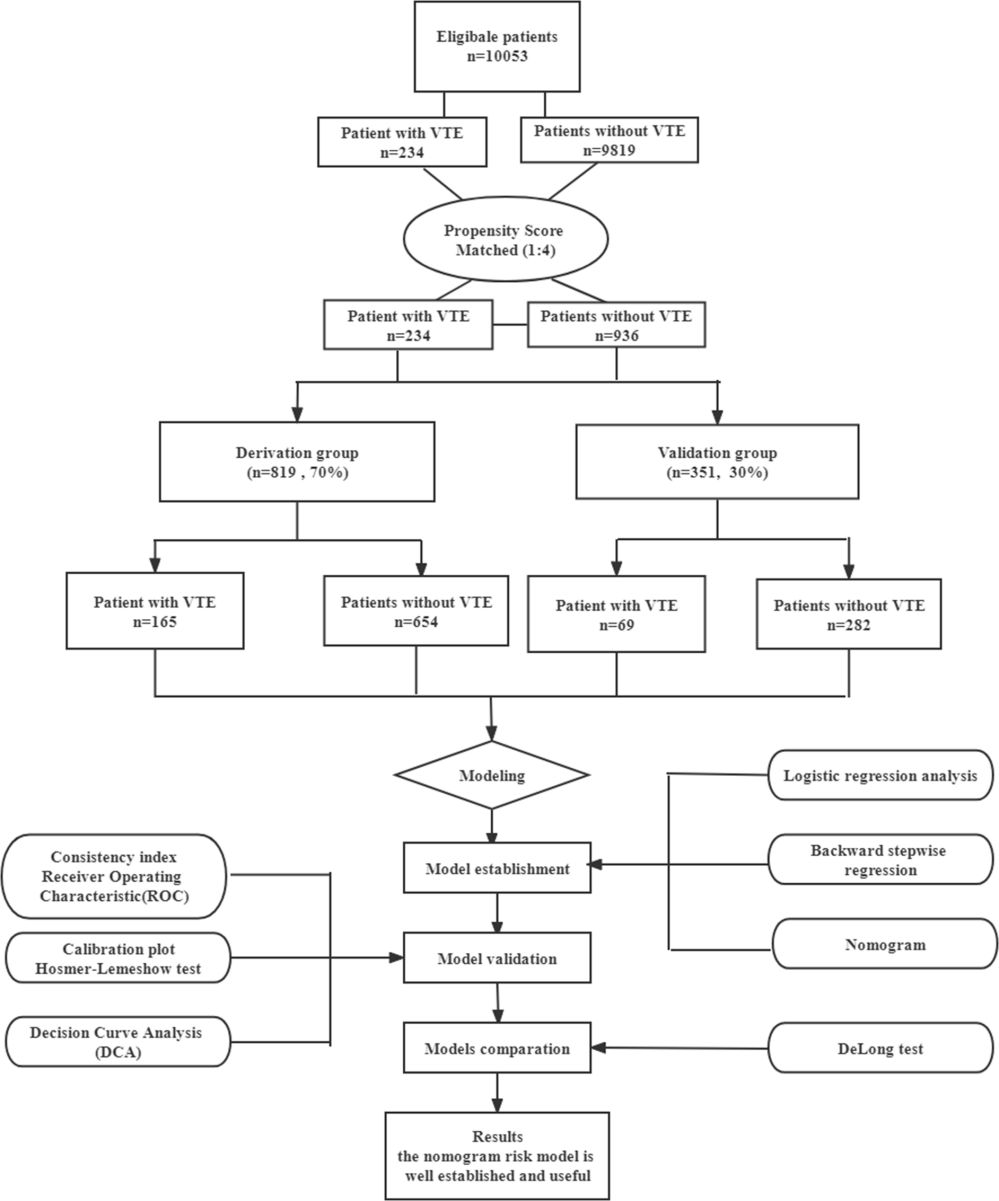
Flow chart of the study design and analysis. VTE, venous thromboembolism.

This study was a matched case-control study. Data from a total of 10,053 newly diagnosed lung cancer patients admitted to The Affiliated Tumor Hospital of Xinjiang Medical University between January 2016 and December 2021 were collected and retrospectively analyzed. Patients enrolled in our study were all inpatients. The calculation of the sample size was based on demonstrating the probability of exposure among sampled control patients was 0.2 with 90% power and 5% statistical significance. Therefore, the obtained sample size of 234 VTE patients was adequate to address the study aims. To reduce potential selection bias between groups, a 1:4 ratio propensity score matching (PSM) method was performed with optimal full matching (22) with the covariates age, gender, and ethnicity. For each VTE patient, a matched sample of 4 non-VTE patients was also obtained. Thus, this sample of 936 non-VTE patients were selected out of the total 9819 control cases. Enrolled patients were further randomized into the derivation group (70%, 165 VTE patients and 654 non-VTE patients) and the validation group (30%, 69 VTE patients and 282 non-VTE patients) (**Figure 1**).

The study was conducted in accordance with the ethical standards revised in the 2013 Declaration of Helsinki. This study was approved by the Ethics Committee of The Affiliated Tumor Hospital of Xinjiang Medical University with the ethical approval number: 2019BC007. However, the requirement to obtain informed consent for any research utilizing patients’ medical information was waived owing to the retrospective design of the study.

### Patients and eligibility

The inclusion criteria for the VTE group were as follows: (a) 18 years or older; (b) length of hospital stay >3 days;(c) all primary lung malignant tumors confirmed by histopathological examination; (d) with DVT and/or PE events confirmed by objective imaging methods and (f) complete case data. The primary diagnosis of VTE (DVT and/or PE) and comorbidities were abstracted from electronic medical records (EMR) according to the International Statistical Classification of Diseases, 10^th^ Clinical Modification (ICD-10 CM).

The exclusion criteria were as follows: (a) inpatients hospitalized for<72h; (b) second malignancy other than lung cancer; (c) patients with acute coronary syndrome or history of implantation of intracardiac devices (pacemakers, prosthetic valves, or implantable cardioverter-defibrillators, etc.) or history of VTE prior to admission; (d) prophylactic anticoagulation before VTE occurring during antitumor therapies; (e) patients receiving long-term therapeutic anticoagulation (at least 3 months) before hospitalization; (f) previous hematological diseases, rheumatoid arthritis, limited liver and kidney function and/or damage.

Controls were selected by propensity score matching (PSM) method from adult lung cancer inpatients (length of hospital stay>3 days) admitted into the same departments during the same period as cases, without an ICD-10 code for VTE (DVT and/or PE) at discharge. The same exclusion criteria used for cases were also applied to controls. Controls were frequency-matched to cases at a ratio of 4:1.

### VTE diagnosis

Symptomatic or incidental VTE that occurred within the first 6 months of cancer diagnosis during the patients’hospitalization was the primary outcome of the study, including DVT and PE. The diagnosis of PE was made by computed tomography pulmonary angiography(CTPA) according to the consensus guidelines, with single/bilateral/multi-lobar pulmonary artery embolism and its branches being the main type. The diagnosis of DVT was made by computed tomography(CT) angiography or complete compression venous ultrasonography(CUS) according to standard ultrasonographic criteria (23), with venous blood stasis in the upper- and lower-extremities. All VTE events were was independently reviewed by two experienced experts in the field of angiology and radiology.

### Data collection and follow-up

The abstracted data were extracted from the electronic EMR retrospectively: patients’ demographic and clinicopathological characteristics including age, gender, ethnicity, smoking history, blood type, body mass index (BMI) before initial treatment (baseline), Eastern Cooperative Oncology Group performance status (ECOG PS), central venous catheters(CVCs) indwelling history, tumor pathology(adenocarcinoma and non-adenocarcinoma) and clinical stage(early and advanced stage), PD-L1 expression(<50%, ≥50%), and diver genes status(EGFR and KRAS mutation, ALK and ROS1 rearrangement and wild type); Detailed information about historic treatment regimens including surgery, targeted therapy, chemotherapy, radiotherapy or immunotherapy during the follow-up period for case group and control group; Comorbid conditions including hypertension, diabetes and coronary heart disease. In order to avoid the effect of anticancer treatment on the value of the indicator, all laboratory examination data were obtained from the pre-treatment baseline assessment after admission. All D-dimer levels were assayed in plasma using the Innovance D-dimer immunoturbidimetric method (Siemens Healthcare, Eschborn, Germany). Laboratory examination data including routine blood indicators(haemoglobin(Hb), leucocyte platelet(Plt), neutrophil-to-lymphocyte ratio(NLR), platelet-to-lymphocyte ratio(PLR)); Coagulation function indexs(prothrombin time(PT), activated partial thromboplastin time(APTT), fibrinogen (Fbg), D-dimer (Ddi)); Biochemical routine (albumin, alanine transaminase(ALT), aspartate transaminase(AST), Lactate dehydrogenase (LDH), triglyceride(TG)); Pro-Brain natriuretic peptide (pro-BNP)) and tumor biomarkers(cytokeratin-19-fragment(CYFR121-1), carcinoembryonic antigen(CEA), carbohydrate antigen 125(CA125), Gastrin releasing peptide(Pro-GRP) and neuron-specific enolase(NSE)). Further, VTE risk was evaluated *via* Khorana Prediction Score, Padua Prediction Score, Caprini Risk Assessment model and COMPASS-cancerassociated thrombosis score (COMPASS-CAT), respectively. Predictor variables were identified from synthesis of the literatures about VTE risk (24–27). All patients were followed up by telephone or hospital visit until the occurrence of VTE, death or end of follow-up in March 2022.

### Derivation and internal verification of the nomogram

The risk assessment model was developed in the derivation cohorts by binary multiple logistic regression analysis. Internal validation was performed in the internal validation cohorts. The Chi-square test for categorical variables were used to compare the baseline characteristics between the derivation and validation cohorts.

Logistic regression analysis for univariate and multivariate analyses and stepwise regression analysis were used to evaluate the independent factors influencing thrombosis in lung cancer patients. Variables with a *P*-value <0.05 in the univariate regression analysis were included in multivariate logistic regression analysis. Afterward, variables with clinical significance and those with *P* < 0.05 in the multivariate analysis were included in the backward stepwise logistic regression analysis. Backward stepwise selection was applied using the likelihood ratio test with Akaike’s information criterion (AIC) minimum method as the termination rule (28). The effect measure of each variable on VTE was presented as odds ratios (OR) and corresponding 95% confidence intervals (CI). Variance Inflation Factor (VIF) was used for collinearity diagnosis of the covariates in the model. Then the nomogram was constructed by using the RMS package in the R (r4.1.3) software to visually score individual risk probabilities of VTE in lung cancer patients.

The reliability of internal validation was assessed using the bootstrap method with 1000 replicates. The discrimination of the nomogram model was evaluated by the consistency index (C-index) and receiver operating characteristic curve (ROC) analysis. Further, the area under the ROC curve (AUC) was obtained to quantitatively evaluate the discriminative ability of the nomogram to predict VTE in patients with lung cancer. The possible value for an AUC ranges from 0.5 (no better discrimination than chance) to 1.0 (perfect discrimination). Moreover, calibration curve was plotted to assess the calibration of the nomogram with the Hosmer-Lemeshow goodness-of-fit test, and a p-value of the Hosmer-Lemeshow test > 0.05 indicates that a model has high goodness of fit. Finally, decision curve analysis (DCA) was performed to assess the clinical utility of the predictive nomogram model for guiding clinical decision making of thromboprophylaxis in lung cancer (29).

### Assessment of risk of bias and applicability

Risk of bias (ROB) and applicability was assessed using the Prediction Study Risk Of Bias Assessment Tool (PROBAST) (30). The assessment of ROB comprises four domains—participants, predictors, outcome, and analysis, questions are answered as “yes”, “probably yes”, “probably no”, “no”, or “no information”. The degree of ROB and applicability were judged as “low”, “high”, or “unclear” for each domain. Risk of bias and applicability assessment was performed by one reviewer and checked by a further reviewer. Any disagreements were mediated by a third reviewer.

### Diagnostic value of the nomogram

The diagnostic performance of new prediction model and current models (Khorana, Caprini, Padua and COMPASS-CAT) were evaluated assessed by calculating the AUC. The diagnostic value of the nomogram was assessed by calculating the sensitivity, specificity, positive predictive values (PPV) and negative predictive values(NPV). Further, the comparison of new prediction model with current models was performed by comparing ROC curves using the DeLong’s test.

### Statistical analysis

SPSS version 25.0 software (IBM, USA) and R version 4.1.3 software (https://www.r-project.org/) were performed for statistical analysis. PSM was performed with optimal full matching by the R package ‘*Matchlt*’. Multiple imputation with chained equations was used to replace missing data for BMI values. Considering the model’s extrapolation accuracy and clinical application, continuous variables were transformed into categorical variables by determining the optimal cut-off (OCF) value according to the maximum Youden index on the basis of the receiver operating characteristic(ROC) curves. The continuous BMI variable was categorized based on cut-off values routinely used in clinical practice for ease of interpretation. Categorical variables were presented as whole numbers and proportions. Comparisons between two groups were performed using the Chi-squared test for categorical variables. A two- sided p-value < 0.05 indicated statistical significance.

## Results

### Patient characteristics and VTE incidence

A total of 1170 patients were enrolled and randomly assigned at a ratio of 7:3, resulting in 819 patients assigned to the derivation group and 351 assigned to the validation group (**Figure 1**). Based on the current sample size and effect size, our study has a statistical power of 91%, which exceeds the minimal statistical power of 80% required for the adequacy of sample sizes. The control’s lung cancer duration(time since cancer diagnosis) was ≥ the case’s duration to ensure that the control would have equal exposure to the risk of VTE induced by cancer. The demographic and clinical characristics of patients in the derivation and validation cohorts are illustrated in **Table 1**, indicate that most characterisics were similarly distirbuted between the two cohorts. Overall, 165(20.1%) patients in the development cohort and 69(19.7%) patients in the validation cohort developed VTE, and there was no significant difference in VTE morbidity between the two cohorts (c²=0.037, *P* = 0.848). Similarly, there were no significant differences of incidence in DVT alone(4.2% vs.3.1%), PE alone (14.9% vs.15.4%), and DVT&PE (1.1% vs.1.1%)(c²=0.407, 0.046, 0.004, *P=*0.688, 0.830, 0.951, respectively) (**Table 1**)

**Table 1:** Demographic and clinical characteristics of patients in derivation and validation cohorts.

Variables [n (%)]	Categories	Development group				Validation group			
		VTE (+)	VTE (-)	χ²	*P* value	VTE (+)	VTE (-)	χ²	*P* value
**Age (years)**				—	0.770*			—	0.699*
<40		2 (1.2)	17 (2.6)			2 (2.9)	6 (2.1)		
40-50		11 (6.7)	40 (6.1)			6 (8.7)	17 (6.0)		
50-60		42 (25.5)	176 (26.9)			21 (30.4)	78 (27.7)		
≥60		110 (66.7)	421 (64.4)			40 (58.0)	181 (64.2)		
**Sex**				0.057	0.817			0.645	0.422
Male		89 (53.9)	362 (55.4)			42 (60.9)	154 (54.6)		
Female		76 (46.1)	292 (44.6)			27 (39.1)	128 (45.4)		
**Ethnicity**				0.857	0.651			3.006	0.222
Han		138 (83.6)	528 (80.7)			53 (76.8)	233 (82.6)		
Uygur		15 (9.1)	65 (9.9)			7 (10.1)	30 (10.6)		
Others ethnic minorities		12 (7.3)	61 (9.3)			9 (13.0)	19 (6.7)		
**VTE events type**				—	—			—	—
DVT alone		34 (20.6)				11 (15.9)			
PT alone		122 (73.9)				54 (78.3)			
DVT&PT		9 (5.5)				4 (5.8)			
**Blood type**				28.881	<0.001			—	0.021*
A		39 (28.7)	122 (25.4)			17 (32.7)	56 (27.9)		
B		55 (40.4)	107 (22.3)			17 (32.7)	38 (18.9)		
AB		18 (13.2)	59 (12.3)			6 (11.5)	17 (8.5)		
O		24 (17.6)	192 (40.0)			12 (23.1)	90 (44.8)		
BMI^a^ (kg/m^2^)				9.363	0.009			6.986	0.030
Normal(<24.0)		72 (43.6)	370 (56.6)			25 (36.2)	146 (51.8)		
Overweight(24.0-28.0)		69 (41.8)	220 (33.6)			33 (47.8)	89 (31.6)		
Obesity (≥28.0)		24 (14.5)	64 (9.8)			11 (15.9)	47 (16.7)		
**ECOG PS**				2.189	0.139			0.987	0.320
0-1		159 (96.4)	607 (92.8)			66 (95.7)	257 (91.1)		
≥2		6 (3.6)	47 (7.2)			3 (4.3)	25 (8.9)		
**Histology**				15.449	<0.001			10.908	0.001
Adenocarcinoma		45 (27.3)	291 (44.5)			22 (31.9)	155 (55.0)		
Non-adenocarcinoma		120 (72.7)	363 (55.5)			47 (68.1)	127 (45.0)		
cTNM stage^b^				12.687	<0.001			13.715	<0.001
I-II		25 (15.2)	191 (29.2)			5 (7.2)	84 (29.8)		
III-IV		140 (84.8)	463 (70.8)			64 (92.8)	198 (70.2)		
**Smoke history**				0.005	0.943			8.239	0.004
Never		96 (58.2)	376 (57.5)			26 (37.7)	163 (57.8)		
Current and former		69 (41.8)	278 (42.5)			43 (62.3)	119 (42.2)		
**CVC history**		103 (62.4)	148 (22.6)	96.303	<0.001	42 (60.9)	72 (25.5)	29.976	<0.001
**History of disease**									
Hypertension		66 (40.0)	179 (27.4)	9.431	0.002	18 (26.1)	91 (32.3)	0.722	0.395
Diabetes mellitus		17 (10.3)	96 (14.7)	1.769	0.183	9 (13.0)	51 (18.1)	0.67	0.413
Heart disease		15 (9.1)	60 (9.2)	<0.001	1	8 (11.6)	41 (14.5)	0.193	0.661
**Blood routine**									
Hemoglobin(g/L)				2.963	0.085			—	0.190*
	<95	5 (3.0)	46 (7.0)			2 (2.9)	23 (8.2)		
	≥95	160 (97.0)	608 (93.0)			67 (97.1)	259 (91.8)		
Platelet(×109 g/L)				2.627	0.105			10.221	0.001
	<222	73 (44.2)	338 (51.7)			22 (31.9)	153 (54.3)		
	≥222	92 (55.8)	316 (48.3)			47 (68.1)	129 (45.7)		
NLR				9.726	0.002			4.987	0.026
	<3.42	64 (38.8)	345 (52.8)			25 (36.2)	147 (52.1)		
	≥3.42	101 (61.2)	309 (47.2)			44 (63.8)	135 (47.9)		
PLR				1.212	0.271			0.914	0.339
	<146.24	58 (35.2)	263 (40.2)			22 (31.9)	110 (39.0)		
	≥146.24	107 (64.8)	391 (59.8)			47 (68.1)	172 (61.0)		
**Blood coagulation**									
D-dimer(mg/L)				98.756	<0.001			64.215	< 0.001
	<2.06	58 (35.2)	497 (76.0)			20 (29.0)	224 (79.4)		
	≥2.06	107 (64.8)	157 (24.0)			49 (71.0)	58 (20.6)		
APTT(sec)				10.083	0.001			6.346	0.012
	<24.15	29 (17.6)	57 (8.7)			13 (18.8)	22 (7.8)		
	≥24.15	136 (82.4)	597 (91.3)			56 (81.2)	260 (92.2)		
PT(sec)				26.946	<0.001			21.062	< 0.001
	<11.45	44 (26.7)	324 (49.5)			11 (15.9)	133 (47.2)		
	≥11.45	121 (73.3)	330 (50.5)			58 (84.1)	149 (52.8)		
Fbg(g/L)				5.045	0.025			5.226	0.022
	<3.33	63 (38.2)	316 (48.3)			20 (29.0)	127 (45.0)		
	≥3.33	102 (61.8)	338 (51.7)			49 (71.0)	155 (55.0)		
**Biochemical routine**									
Albumin(g/L)				8.02	0.005			8.541	0.003
	<41	123 (74.5)	408 (62.4)			53 (76.8)	160 (56.7)		
	≥41	42 (25.5)	246 (37.6)			16 (23.2)	122 (43.3)		
ALT(IU/L)				0.666	0.414			0.265	0.607
	<9.05	25 (15.2)	81 (12.4)			10 (14.5)	32 (11.3)		
	≥9.05	140 (84.8)	573 (87.6)			59 (85.5)	250 (88.7)		
AST(IU/L)				1.509	0.219			2.218	0.136
	<18.05	70 (42.4)	241 (36.9)			31 (44.9)	97 (34.4)		
	≥18.05	95 (57.6)	413 (63.1)			38 (55.1)	185 (65.6)		
LDH(U/L)				12.007	0.001			8.629	0.003
	<258.25	112 (67.9)	528 (80.7)			46 (66.7)	235 (83.3)		
	≥258.25	53 (32.1)	126 (19.3)			23 (33.3)	47 (16.7)		
TG(mmol/L)				5.35	0.021			2.526	0.112
	<1.37	79 (47.9)	381 (58.3)			36 (52.2)	179 (63.5)		
	≥1.37	86 (52.1)	273 (41.7)			33 (47.8)	103 (36.5)		
Pro-BNP(pg/ml)				15.727	<0.001			11.3	0.001
	<263.75	113 (68.5)	541 (82.7)			46 (66.7)	240 (85.1)		
	≥263.75	52 (31.5)	113 (17.3)			23 (33.3)	42 (14.9)		
**Tumor biomarkers**									
CYFA21-1(ng/mL)				3.392	0.066			0.003	0.298
	<3.34	92 (55.8)	418 (63.9)			39 (56.5)	181 (64.2)		
	≥3.34	73 (44.2)	236 (36.1)			30 (43.5)	101 (35.8)		
CEA(ug/L)				4.186	0.041			4.267	0.039
	<3.18	58 (35.2)	290 (44.3)			27 (39.1)	152 (53.9)		
	≥3.18	107 (64.8)	364 (55.7)			42 (60.9)	130 (46.1)		
CA125(U/mL)				10.858	0.001			9.77	0.002
	<38.45	82 (49.7)	419 (64.1)			33 (47.8)	194 (68.8)		
	≥38.45	83 (50.3)	235 (35.9)			36 (52.2)	88 (31.2)		
Pro-GPR(pg/mL)				3.974	0.046			4.117	0.042
	<37.23	112 (67.9)	386 (59.0)			49 (71.0)	160 (56.7)		
	≥37.23	53 (32.1)	268 (41.0)			20 (29.0)	122 (43.3)		
NSE(ng/mL)				1.273	0.259			0.478	0.49
	<17.32	100 (60.6)	362 (55.4)			41 (59.4)	152 (53.9)		
	≥17.32	65 (39.4)	292 (44.6)			28 (40.6)	130 (46.1)		
**Molecular driver**									
EGFR mutation		21 (13.5)	115 (18.5)	1.748	0.186	9 (13.4)	48 (18.3)	0.563	0.453
KRAS mutation		13 (8.4)	34 (5.7)	1.129	0.288	5 (7.8)	16 (6.3)	–	0.585*
ROS1 rearrangement		49 (31.2)	103 (16.5)	16.459	<0.001	49 (31.2)	103 (16.5)	21.308	<0.001
ALK rearrangement		28 (17.8)	104 (16.6)	0.069	0.793	10 (14.7)	31 (11.4)	0.293	0.588
PD-L1 expression(≥50%)		39 (6.7)	19 (12.8)	5.146	0.023	24 (9.3)	9 (14.8)	1.027	0.311
Treatment history^c^									
Chemotherapy		93 (56.4)	186 (28.4)	44.504	<0.001	45 (65.2)	93 (33.0)	22.817	<0.001
Radiotherapy		54 (32.7)	125 (19.1)	13.513	<0.001	15 (21.7)	52 (18.4)	0.206	0.650
Targeted Therapy		123 (18.8)	33 (20.0)	0.057	0.812	49 (17.4)	23 (33.3)	7.707	0.006
Immunotherapy		18 (10.9)	33 (5.0)	6.785	0.009	6 (8.7)	23 (8.2)	<0.001	1
Surgery		56 (33.9)	192 (29.4)	1.102	0.294	20 (29.0)	72 (25.5)	0.187	0.666
**The Khorana score**				4.056	0.044			3.841	0.050
1-2 points		142 (86.1)	599 (91.6)			56 (81.2)	255 (90.4)		
≥3 points		23 (13.9)	55 (8.4)			13 (18.8)	27 (9.6)		

a BMI was categorized according to the Chinese population standards.

b According to the 8 th edition of the AJCC/UICC staging system.

c All the anti-cancer therapies were within 6 month before the VTE diagnosis.

*P values were derived from Fisher Exact test.

VTE, venous thromboembolism; BMI, Body Mass Index; ECOG PS, Eastern Cooperative Oncology Group performance status; CVC, central venous catheter; NLR, Neutrophil-to-Lymphocyte ratio; PLR, Platelet-to-Lymphocyte ratio; PT, prothrombin time; APTT, activated partial thromboplastin time; Fbg, fibrinogen; ALT, alanine transaminase; AST, aspartate transaminase; LDH, Lactate dehydrogenase;TG, triglyceride; pro-BNP, Pro-Brain natriuretic peptide; CYFR121-1, cytokeratin-19-fragment; CEA, carcinoembryonic antigen; CA125, carbohydrate antigen 125; Pro-GRP, Gastrin releasing peptide; NSE, neuron-specific enolase.

### Exploration of risk factors for VTE

The results of univariate and multivariate logistic regression analyses based on the factors associated with VTE are presented in **Table 2**. In the validation group, univariate analysis showed that the following factors were statistically significant: BMI, histology, clinical stage, CVC history, hypertension, NLR, coagulation function indexs(D-dimer, ATTT, PT and Fbg levels), biochemical routine indexs(albumin, LDH and TG levels), Pro-BNP, tumor biomarkers(CEA, CA125 and Pro-GPR levels), molecular driver status(ROS1 rearrangement and PD-L1 high expression) and historic treatment regimens(chemotherapy, radiotherapy, and immunotherapy history)(*P*<0.05).

**Table 2:** Univariate and multivariate regression analysis of risk factors associated with VTE in lung cancer.

Variables	Univariate analysis			Multivariate analysis			Stepwise regression analysis		
	β	OR(95% CI)	*P* value	β	OR(95% CI)	*P* value	β	OR(95% CI)	*P* value
**Age (years)**									
<40		Ref							
40-50	0.850	2.34 (0.55-16.16)	0.301						
50-60	0.708	2.03 (0.55-13.11)	0.357						
≥60	0.798	2.22 (0.62-14.14)	0.291						
**Sex**, Female (vs. Male)	0.058	1.06 (0.75-1.49)	0.745						
**Ethnicity**									
Han	Ref	Ref							
Uygur	-0.128	0.88 (0.47-1.56)	0.68						
Others ethnic minorities	-0.288	0.75 (0.38-1.39)	0.389						
**BMI** ^a^ (kg/m2)									
Normal(<24.0)		Ref							
Overweight(24.0-28.0)	0.896	2.45 (1.73-3.51)	<0.001	0.792	2.21 (1.29-3.81)	0.004	0.643	1.90 (1.19-3.07)	0.008
Obesity (≥28.0)	0.854	2.35 (1.37-4.24)	0.003	0.779	2.18 (0.97-4.84)	0.057	0.659	1.93 (0.95-3.88)	0.066
**ECOG PS**, ≥2 (vs. 0-1)	-0.713	0.49 (0.18-1.08)	0.104						
**Adenocarcinoma**(vs. Non-Adenocarcinoma)	0.761	2.14 (1.48-3.14)	<0.001	1.273	3.57 (2.07-6.37)	<0.001	1.099	3.00 (1.88-4.87)	<0.001
**cTNM stage^b^, III-IV(vs. I-II)**	0.837	2.31 (1.49-3.72)	<0.001	1.328	3.78 (1.98-7.56)	<0.001	1.011	2.75 (1.58-4.96)	0.001
**Smoke history**	0.565	1.76 (1.03-2.93)	0.873						
**CVC history**	1.737	5.68 (3.96-8.21)	<0.001	1.610	5.00( 2.92-8.72)	<0.001	1.534	4.64 (2.86-7.62)	<0.001
**History of disease**									
Hypertension	0.571	1.77 (1.24-2.52)	0.002	0.421	1.52 (0.9-2.58)	0.118			
Diabetes mellitus	-0.400	0.67 (0.37-1.13)	0.147						
Heart disease	-0.010	0.99 (0.53-1.75)	0.974						
Khorana score, ≥3 points(vs. 1-2 points)	-0.713	0.49 (0.18-1.07)	0.032	-0.070	0.93 (0.42-1.98)	0.858			
**Blood routine**									
Hemoglobin count ≥95 g/L	0.844	2.42 (1.04-7.07)	0.065						
Platelet count ≥222×109 /L	0.300	1.35 (0.96-1.9)	0.088						
NLR ≥3.42	0.565	1.76 (1.25-2.51)	0.001	0.239	1.27 (0.74-2.17)	0.381			
PLR ≥146.24	0.215	1.24 (0.87-1.78)	0.234						
**Blood coagulation**									
D-dimer ≥2.06 mg/L	1.765	5.84 (4.06-8.47)	<0.001	1.600	4.96 (2.86-8.74)	<0.001	1.719	5.58 (3.54-8.94)	<0.001
APTT ≥24.15 sec	-0.799	0.45 (0.28-0.73)	0.001	-0.373	0.69 (0.33-1.49)	0.337			
PT ≥11.45 sec	1.215	3.37 (2.3-5.04)	<0.001	0.716	2.05 (1.17-3.62)	0.012	0.766	2.15 (1.32-3.54)	0.002
Fbg ≥3.33 g/L	0.412	1.51 (1.07-2.15)	0.02	0.717	2.05 (1.21-3.51)	0.008	0.563	1.76 (1.12-2.78)	0.015
**Biochemical routine**									
Albumin ≥41 g/L	-0.562	0.57 (0.38-0.83)	0.004	0.213	1.24 (0.66-2.30)	0.501			
ALT ≥9.05 IU/L	-0.236	0.79 (0.49-1.31)	0.345						
AST≥18.05 IU/L	-0.236	0.79 (0.56-1.12)	0.188						
LDH ≥258.25 U/L	0.683	1.98 (1.35-2.89)	<0.001	-0.005	0.99 (0.55-1.77)	0.986			
TG ≥1.37 mmol/L	0.419	1.52 (1.08-2.14)	0.017	0.808	2.24 (1.33-3.83)	0.003	0.631	1.88 (1.19-2.99)	0.007
Pro-BNP ≥263.75 pg/ml	0.788	2.20 (1.49-3.23)	<0.001	0.606	1.83 (0.99-3.40)	0.054			
**Tumor biomarkers**									
CYFA21-1 ≥3.34ng/mL	0.344	1.41 (0.99-1.99)	0.054						
CEA ≥3.18 ug/L	0.385	1.47 (1.03-2.11)	0.033	-0.022	0.98 (0.56-1.7)	0.939			
CA125 ≥38.45 U/mL	0.588	1.80 (1.28-2.55)	0.001	-0.008	0.99 (0.57-1.71)	0.977			
Pro-GPR ≥37.23 pg/mL	-0.386	0.68 (0.47-0.97)	0.038	-0.342	0.71 (0.42-1.18)	0.193			
NSE ≥17.32 ng/mL	-0.211	0.81 (0.57-1.14)	0.224						
**Molecular driver**									
EGFR mutation	-0.371	0.69 (0.41-1.12)	0.151						
KRAS mutation	0.491	1.52 (0.76-2.9)	0.214						
ROS1 rearrangement	0.833	2.30 (1.54-3.41)	<0.001	0.968	2.63 (1.5-4.62)	0.001	1.055	2.87 (1.74-4.75)	<0.001
ALK rearrangement	0.086	1.09 (0.68-1.71)	0.703						
PD-L1 expression(≥50%)	0.713	2.04(1.12-3.6)	0.016	0.961	2.61 (0.8-8.05)	0.101			
**Treatment history** ^c^									
Chemotherapy	1.179	3.25 (2.29-4.63)	<0.001	0.604	1.83 (1.04-3.22)	0.036	0.505	1.66 (1.01-2.7)	0.043
Radiotherapy	0.723	2.06 (1.40-3.00)	<0.001	0.762	2.14 (1.19-3.87)	0.011	0.674	1.96 (1.17-3.29)	0.011
Targeted Therapy	0.077	1.08 (0.69-1.64)	0.727						
Immunotherapy	0.833	2.30 (1.24-4.16)	0.007	-0.082	0.92 (0.28-3.07)	0.894			
Surgery	0.215	1.24 (0.86-1.77)	0.253						

a BMI was categorized according to the Chinese population standards.

b According to the 8 th edition of the AJCC/UICC staging system.

c All the anti-cancer therapies were within 6 month before the VTE diagnosis.

VTE, venous thromboembolism; BMI, Body Mass Index; ECOG PS, Eastern Cooperative Oncology Group performance status; CVC, central venous catheter; NLR, Neutrophil-to-Lymphocyte ratio; PLR, Platelet-to-Lymphocyte ratio; PT, prothrombin time; APTT, activated partial thromboplastin time; Fbg, fibrinogen; ALT, alanine transaminase; AST, aspartate transaminase; LDH, Lactate dehydrogenase;TG, triglyceride; pro-BNP, Pro-Brain natriuretic peptide; CYFR121-1, cytokeratin-19-fragment; CEA, carcinoembryonic antigen; CA125, carbohydrate antigen 125; Pro-GRP, Gastrin releasing peptide; NSE, neuron-specific enolase; OR, odds ratio; CI, confidence interval.

**Figure 2:**
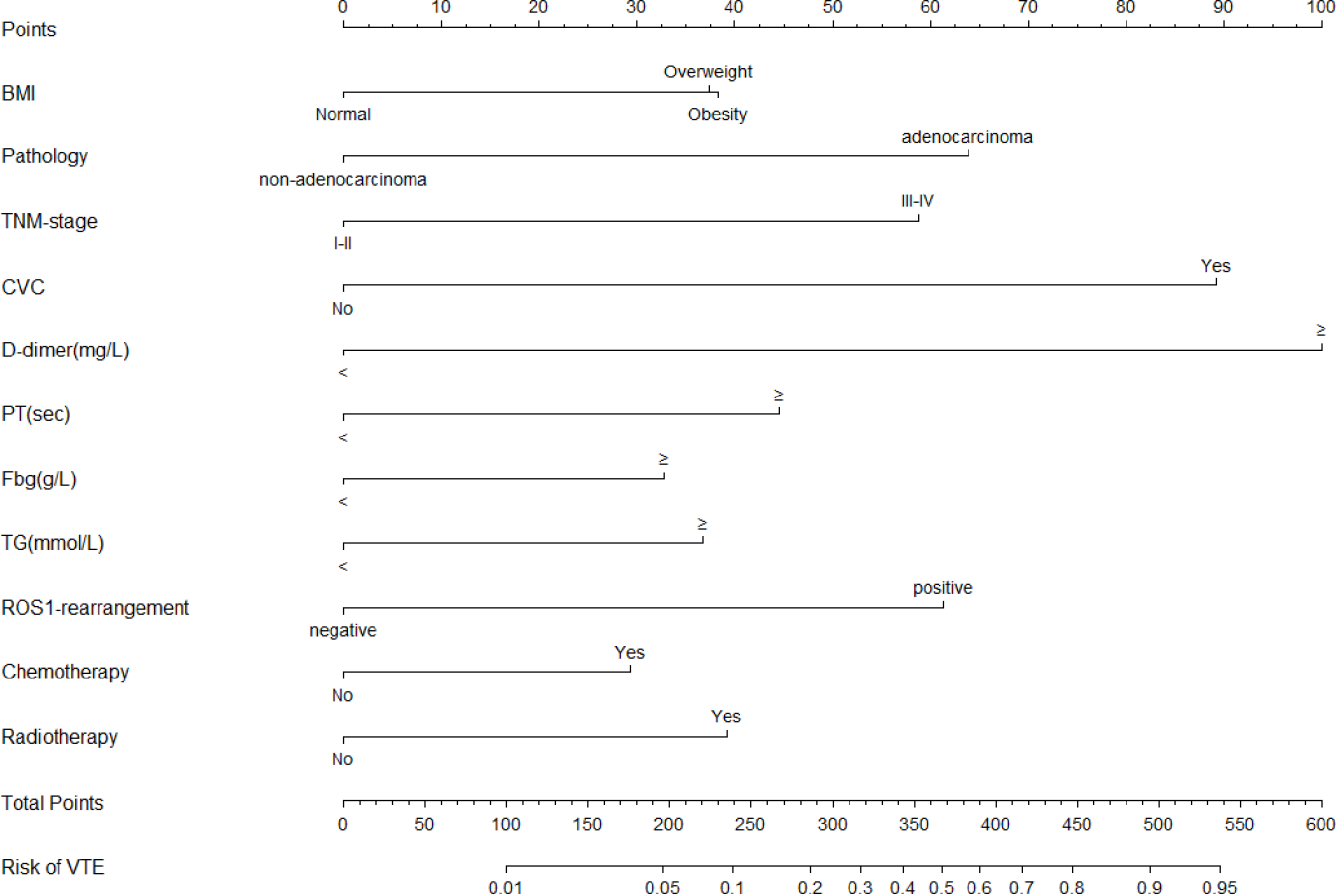
The Nomogram model for prediction of VTE in hospitalized patients with lung cancer. VTE, venous thromboembolism; BMI, Body Mass Index; CVC, central venous catheter; PT, prothrombin time; Fbg, fibrinogen; TG, triglyceride.

Afterward, according to the multivariate and backward stepwise logistic analysis, the results showed overweight(24-28) defined by BMI[1.90(1.19-3.07)], adenocarcinoma[3.00(1.88-4.87)], stageIII-IV[2.75(1.58-4.96)], CVC history[4.64(2.86-7.62)], D-dimer levels≥2.06 mg/L[5.58(3.54-8.94)], PT levels≥11.45sec[2.15(1.32-3.54)], Fbg levels≥3.33 g/L[1.76(1.12-2.78)], TGlevels≥1.37mmol/L[1.88(1.19-2.99)],ROS1rearrangement[2.87(1.74-4.75)],chemotherapy history[1.66(1.01-2.70)] and radiotherapy history[1.96(1.17-3.29)] ROS1rearrangementwere considered to be independent risk factors for VTE in lung cancer, and these factors were eventually incorporated into the final model (**Table 2**). Furthermore, the collinearity diagnostic analysis demonstrated that the VIFs of those risk factors were less than 4, indicating that there is no strong indication of multicollinearity among variables. Thus, there were eleven variables included in the final multivariable prediction model as predictors (**Figure 2**).

### Development of the nomogram model

Based on the regression coefficient of these risk factors, the risk score model of VTE[Logit (P)] was constructed as Logit(P)  =  -6.306+0.643×BMI+1.099×Pathology+1.011×TNM-stage+ 1.543×CVC history+1.719×D-dimer+0.766×PT+0.563×Fbg+0.631×TG+1.055×ROS1-rearrangement+0.505×chemotherapy history+0.674×radiotherapy history. For visualization and convenient clinical use of the predictive model, the mathematical risk prediction model was visualized as a nomogram to predict the likelihood of VTE in hospitalized patients with lung cancer (**Figure 2**). The probability of developing VTE can be determined by assigning points for each variable by drawing a line upward to the Points axis, summing all the points from the variables plotted on the total points axis and then drawing a vertical line from the total points axis straight down to the risk of VTE axis. For example, the application of this model to a 53-year-old patient with lung cancer would show the following results: BMI of 28 kg/m^2^, histology of adenocarcinoma, IV stage, with CVC history, D-dimer of 5.97 mg/L, PT of 11.8 sec, Fbg of 2.48 sec, TG of 1.2g/L, ROS1 rearrangement(+), without chemotherapy and radiotherapy history. The total score of the above predictors was 38 + 64+59+89+100+44+0+0+61+0+0 = 455 and the corresponding risk probability of VTE was 0.81 (81%)

### Performances of discrimination and calibration

**Figure 3:**
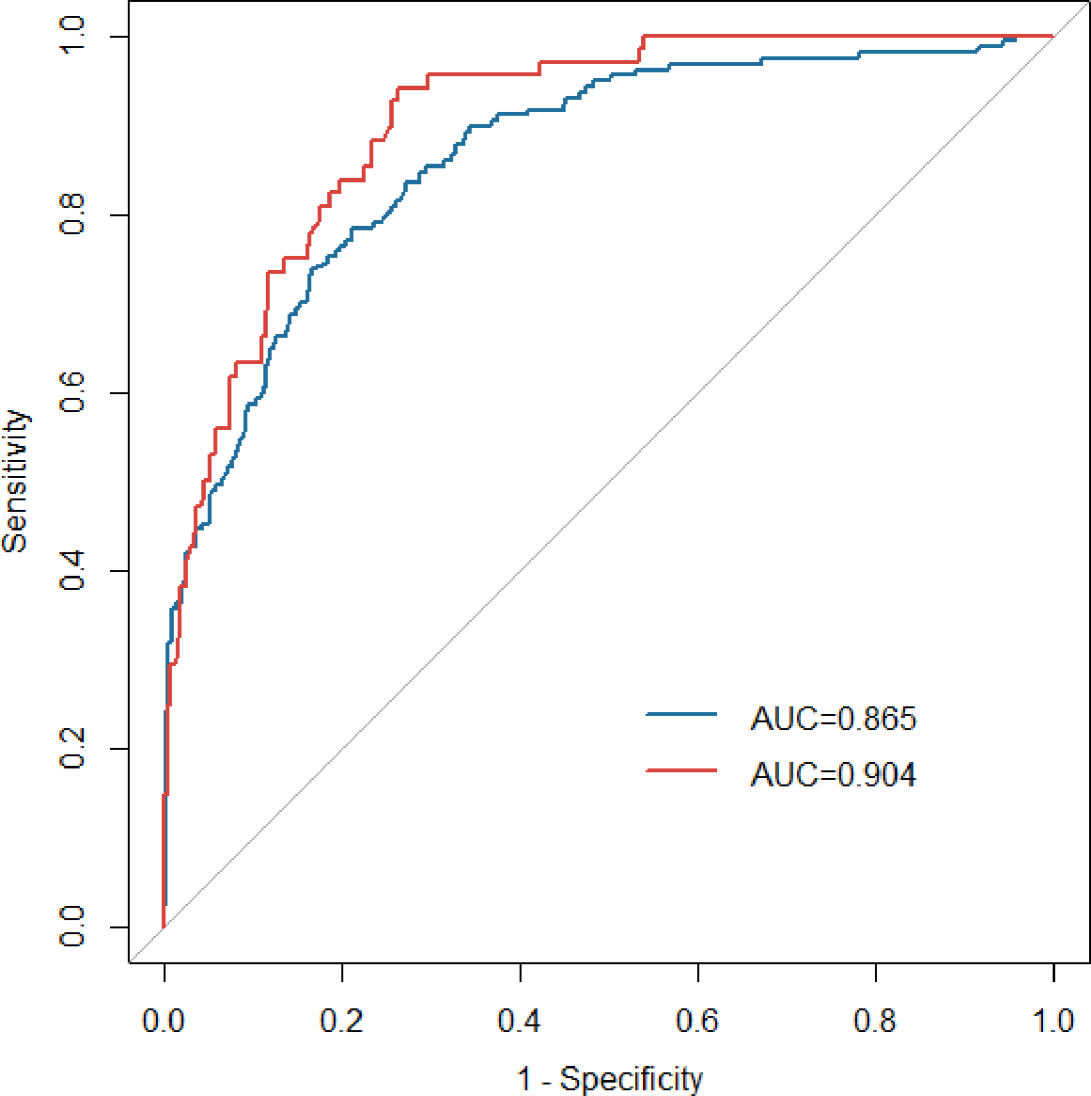
ROC curves of the nomogram in the development (blue line) and validation (red line) groups. ROC, receiver operating characteristic; AUC, area under the curve.

**Figure 4:**
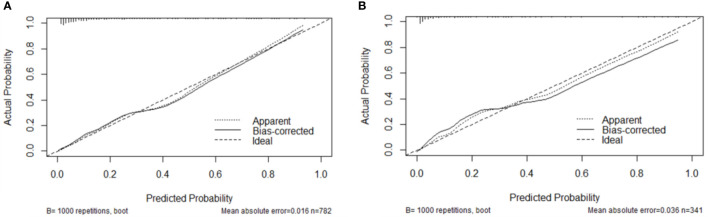
Calibration plots of the nomogram in the development **(A)** and validation **(B)** cohorts.

The AUCs in the development and validation group were 0.865 (95% CI: 0.832-0.897) and 0.904 (95%CI: 0.869-0.939), respectively, which indicated the good prediction performance of the model (**Figure 3**). The proposed nomogram was validated internally using the bootstrap method with 1000-bootstrap repetitions in the development cohort, with a C-index of 0.904, which indicated that the novel proposed model achieved high prediction accuracy. Furthermore, the calibration curve of the nomogram for the prediction of the risk of VTE in patients with lung cancer demonstrated good agreement between prediction and observation in the development (**Figure 4A**) and validation (**Figure 4B**) cohorts. The findings of the Hosmer–Lemeshow goodness-of-fit test also was not significant in the development and validation sets(c²=14.848, 4.276, *P* = 0.062, 0.831, respectively).

### Clinical utility of the model

**Figure 5:**
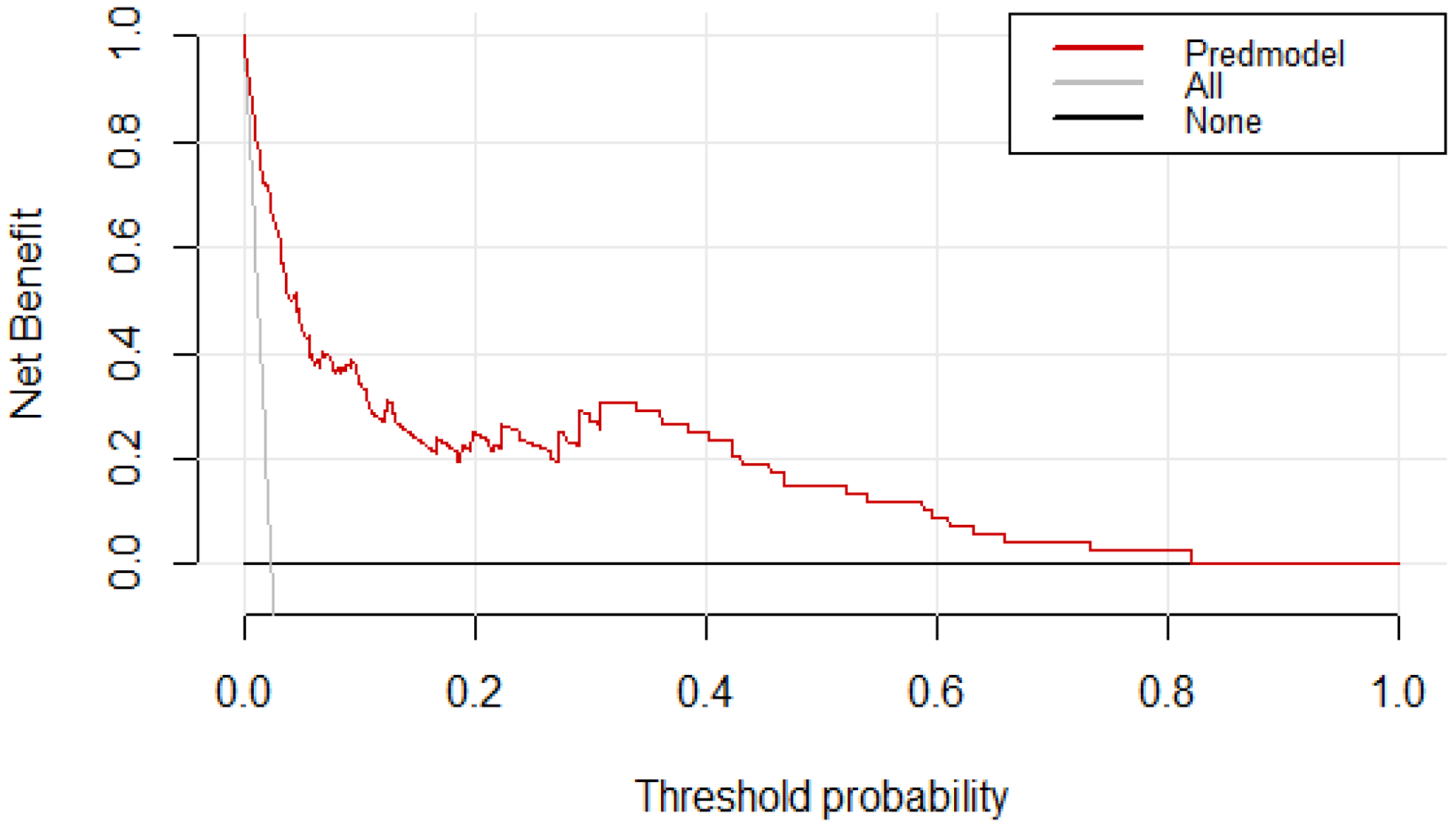
Decision curve analysis (DCA) for assessment of the clinical utility for thromboprophylaxis. The threshold probability represents the predicted risk of VTE for recommending primary thromboprophylaxis.The net benefit balances the risk of VTE with the potential harms of unnecessary thromboprophylaxis and is equal to the true-positive rate minus the weighted false-positive rate.

The DCA curves for the predictive nomogram are presented in **Figure 5**. The clinical utility of nomogram model was estimated using DCA by quantifying the net benefits at different threshold probabilities. The DCA displayed that the nomogram provided superior net benefit of thromboprophylaxis in patients at VTE risk than strategies of treating all and treating none, with a probability threshold interval of 2%–82% (**Figure 5**).

### Risk of bias and applicability

An overview of the risk of bias(ROB) and the applicability for this prediction model is provided in **Supplementary Table 1**. The model was rated as high risk of bias in two domains: Predictors and analysis. The risk model had a high ROB and good applicability. The high risk of bias was judged according to some specific issues in the study design and statistical analysis (see the rationale of rating in **Supplementary Table 1**)

### Comparison with risk assessment models

**Figure 6:**
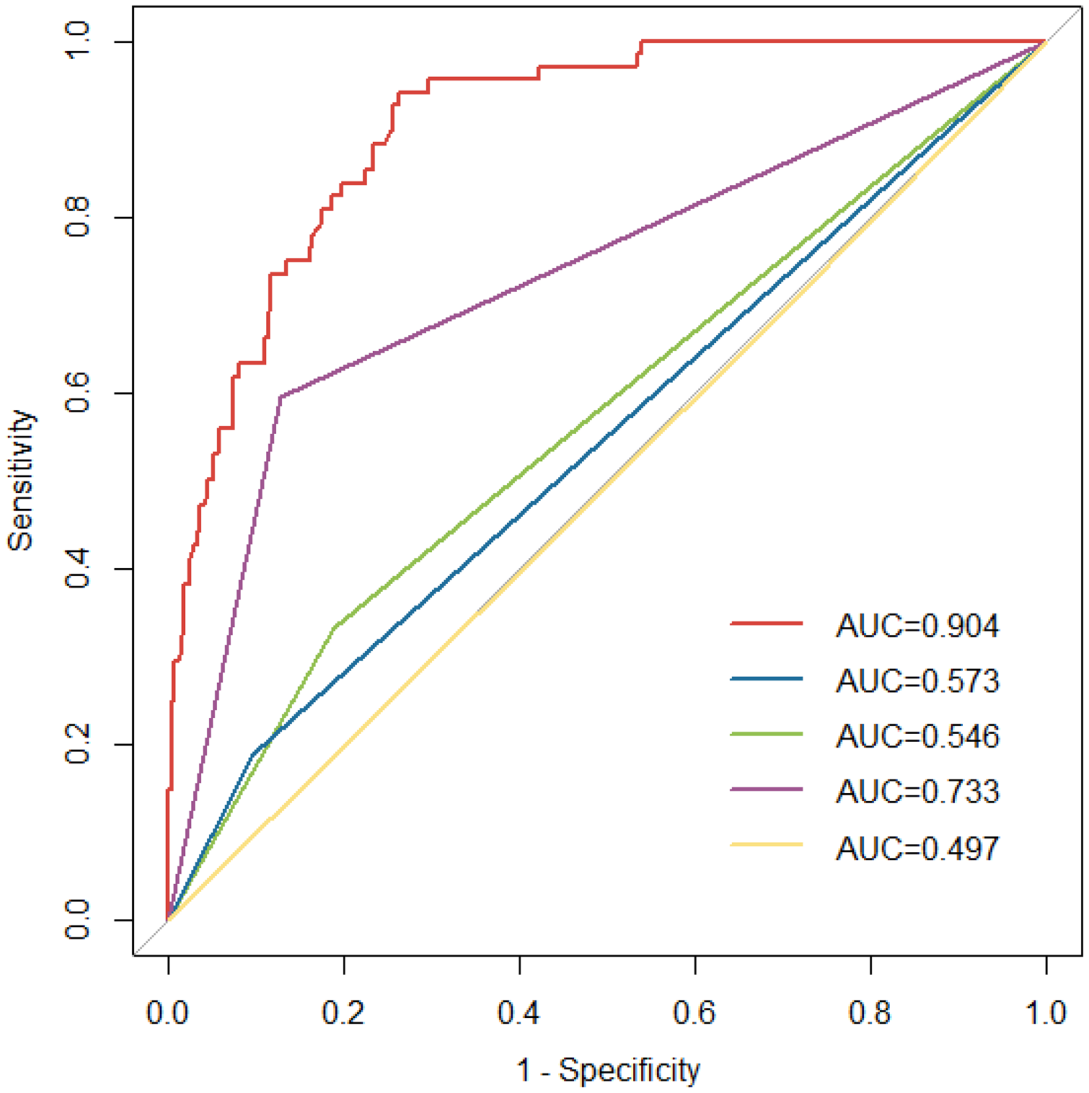
ROC curves for the existing risk assessment models [Khorana score(green line), Caprini risk assessment model(yellow line), Padua score(blue line) and COMPASS-CAT model(purple line)] and nomogram model (red line). ROC, receiver operating characteristic; AUC, area under the curve.

The ROC curves of different VTE risk assessment models are shown in **Figure 6**. The area under the ROC curve for the specific VTE risk-stratification nomogram model (0.904; 95% CI:0.869-0.939) was significantly higher than those of the KRS, Caprini, Padua and COMPASS-CAT models(*Z*=12.087, 11.851, 9.442, 5.340, all *P*<0.001, respectively). Additionally, the risk score of 300.6 was determined as the optimal cutoff value with the maximum Youden index(OR 14.17, 95% CI 9.45-21.64, *P* < 0.001) and was divided into a low-risk group (562 patients with risk score ≤ 300.6) and high-risk group (220 patients with risk score>300.6), respectively. The nomogram model presentedwith a sensitivity (Se) of 73.9%, specificity (Sp) of 83.4%, positive predictive value (PPV) of 52.7% and negative predictive value (NPV) of 92.7%.

## Discussion

Currently, early detection of high-risk factors for cancer patients combined with VTE should be paid for particular attention. Previous studies mainly focused on the analysis of risk factors of lung cancer-associated VTE to establish the risk score system (19–21). However, few studies have been performed for the development of novel nomograms for the prediction of VTE in lung cancer patients, particularly given other factors influencing cancer-associated VTE such as genetic and therapeutic factors (31).Therefore, with a specific focus on both genetic and therapeutic factors, we developed and validated a simple yet highly discriminating, well-calibrated, and parsimonious nomogram prediction model for the occurrence of VTE in hospitalized lung cancer patients in this study, which can provide a theoretical basis for clinical decision-making on thromboprophylaxis on the basis of VTE risk levels. To our knowledge, this is the first to integrate readily obtainable clinical parameters, genetic and therapeutic factors into a modeling of the nomogram for the prediction of VTE in lung cancer. Furthermore, the nomogram prediction model proposed in this study was superior to other established scoring models in risk stratification of VTE patients.

According to the results of the logistic regression analyses, we established a simplified eleven-variables nomogram model, which contains four clinical variables(BMI, histology, clinical stage and CVC history), four biomarkers(D-dimer, PT, Fbg and TG), oncogenic abnormalities(ROS-1 rearrangement) and history of previous radiotherapy and chemotherapy treatment. Many of the risk factors of cancer-associated VTE identified in this analysis, particularly in lung cancer, were mostly consistent with those of the previous literatures (18–21). Among the numerous risk factors, it is widely accepted that higher BMI were patient-related risk factors related to VTE. Several studies have revealed that being obesity or overweight was associated with higher risk of VTE (8, 18, 32), while being underweight was associated with a lower risk of VTE (33). This study confirmed the previous data with a calculated OR of 1.90(95%CI: 1.19-3.07) for overweight. Furthermore, an OR value of 1.93(95%CI: 0.95-3.88) for obesity was observed but was not significant. Considering the body structure, dietary habits and ethnic differences exist between Chinese and Caucasian populations, the Chinese criteria for BMI were used instead of the World Health Organization(WHO) standard for classification in this study. Moreover most studies noted that the modified version of Khorana scale setting the cut-off points of BMI at 24kg/m^2^ for Chinese population could improve the risk stratification and identification of patients with VTE (34). Therefore, we have adjusted the cut-off points of BMI according to the Chinese population standards in our models, for a better evaluation of association between BMI and VTE risk in a Chinese population.

With respect to cancer-related factors, adenocarcinoma was shown to be one of the most powerful predictors for VTE development in our nomogram model. Based on our analysis, the results showed that patients with adenocarcinoma have an approximately 3-fold higher risk of developing VTE than that of non-adenocarcinom apopulation. This result is consistent with the findings of previous studies (19, 21, 35). There is also clear evidence that patients with adenocarcinoma was associated with a markedly higher risk of VTE compared with other pathological types (8, 35). Another recent studies by Tsubata et al. (19) and Li et al. (21) has demonstrated that adenocarcinoma was one of the most powerful predictors for VTE development in the predictive scoring system. These studies led to widespread belief that adenocarcinoma plays a critical role in activating pro-coagulants factors by secreting mucin that may result in thrombus formation. Additionally, we found that patients with advanced stage have a risk of VTE that is 2- to 3-fold higher than those from patients with early stages, which is in accordance with the findings of more recent studies (8, 19, 21, 36, 37). These results suggest that patients with advanced and metastatic cancer has been linked to an increased risk of VTE as compared to tumors that are localized. It is now well acknowledged that high prevalence of VTE occurrence in advanced stage patients may likely be related to the synchronicity of tumor progression and increased coagulation activity (38).

Moreover, most previous studies noted that the use of long-term central venous catheters(CVCs) especially peripheral insertion central catheter (PICC) was strongly associated with increased risk of upper-extremity DVT (39, 40). This study shows that a prior history of CVCs was an independent risk factor for VTE in patients with lung cancer, with an OR of 4.64. This is in agreement with previous studies, which showed CVCs indwelling might be the main cause of upper extremity DVT among cancer patients (8). Furthermore, it should be noted that the incidence of catheter‐related thrombosis (CRT) is closely related to the type of CVC, the thickness of the Catheter and the position of the CVC tip (39–41). This phenomenon might be correlated with vascular endothelial injury and slow blood flow caused by the deep venous indwelling catheter, or compression of the cervical lymph nodes metastasis.

D-dimer is the specific degradation products of crosslinked fibrin and is used as sensitive marker of hypercoagulability status and of endogenous fibrinolysis. It has been established by several studies that D-dimer levels was an independent risk predictor of VTE in various types of cancer (21, 42–44). There is even some evidence suggesting that incorporating D-dimer into the VTE risk score significantly improves VTE risk discrimination and reclassification (45). These results are highly consistent with those from our study, which suggested that D-dimer≥2.06 mg/L can be considered independent risk factors of VTE and can be useful in stratifying risk for VTE in lung cancer. However, it should be noted that the exact D-dimer threshold for the diagnosis of VTE in the models is still controversial. We applied a highly sensitive cut-off of 2.06 mg/L that adapted from the cut-off given by the Youden index, which is similar or higher than these previous studies (20, 42, 43). This may be due to the fact that the tumors of the enrolled patients were mostly at advanced stage, which might resulted in higher baseline D-dimer levels.

Moreover, we have incorporated other noninvasive and obtained easily in clinic indicators into our analyses. The present study found that PT levels≥11.45sec, Fbg levels≥3.33 g/L and TG levels≥1.37mmol/L were an independent risk factor for VTE in patients with lung cancer, with an OR of 2.15, 1.76 and 1.88, respectively. Currently, the association of inflammatory parameters(PLR and NLR) and the risk of thromboembolism has been attracted increasing interest in recent years (46, 47). Based on the logistic regression analysis, our results showed that NLR at baseline were statistically significant in the univariate analysis, whereas elevated NLR were not associated with an increased risk of VTE in the multivariate analysis(*P*=0.381), which is partly compatible with the findings of more recent studies (46, 48). The reason might be related to the effects of neutrophil extracellular traps(NETs) released by tumor-activated neutrophils.

Currently, there is a growing interest in exploring the potential correlation between driver genes and VTE risk in lung cancer (49–51). Clearly, there is a critical need to incorporating positive driver genes into a risk assessment model for lung cancer to improve performance. One of the strengths of our study is the inclusion of molecular drivers in the construction of this model. Interestingly, our results showed that the risk of VTE in ROS1 rearrangements(ROS1+) patients is 2.63-fold greater than that in ROS1- patients, and the odds of VTE in ROS1+ lung cancer were higher than ALK+, EGFR+ and KRAS+ cohorts in the univariate analysis. Similar to our findings, more recent studies by Zhu et al. (50) and Ng et al. (51) have also found that the risk of VTE is significantly increased in patients with ROS+ NSCLC compared to EGFR+ and KRAS+ cases. Although the mechanism is not clear yet, The mucus produced in ROS1 fusions lung adenocarcinoma probably contributes to further platelet recruitment and, consequently, to the thrombus development.

Considering the the treatment-related factors suggested in the literatures including history of chemo-, radiation- and immuno-therapies, several studies have confirmed that chemotherapy, widely used in more than half of the patients, has been reported to be associated with a 2- to 6-fold increased VTE risk (8, 15). By multivariate logistic regression analysis our study showed that having previously undergone radiation or chemotherapy within 6 months before VTE diagnosis was independently associated with an increased risk of VTE for patients with lung cancer, which is in accordance with the findings of Li et al. (21). Plausible explanations for this could be attributed to vascular endothelium damage, reduced endogenous anticoagulant factors(antithrombin, protein C, protein S) and platelet activation (15). Nonetheless, there is less evidence available on the influence of RT on outcome in cancer patients with VTE. Still, a few studies have displayed that there remained a significant correlation between RT and VTE in patients with cancer (16, 52). This may be in part due to the endothelial damage to veins caused by radiation exposure.

To date, many randomized controlled trials(RCTs) have developed several risk assessment models suitable for cancer patients, such as KRS, Caprini, Padua and COMPASS-CAT score. Concerning the comparison of the current model with other existing models, our current data suggest that the area under the ROC curve for the specific VTE risk-stratification nomogram model(0.904; 95% CI:0.869-0.939) was significantly higher than those of the KRS, Caprini, Padua and COMPASS-CAT models. The reason for this differences may have been due to the scale discrimination in the applicable population. KS scale was originally designed for cancer outpatients, which did not include some therapeutic factors (e.g. chemotherapy) occurred during hospitalization. Therefore, the proportion of patients who were at a high risk of VTE may be underestimated. Additionally, Caprini scale has a good predictive value in cancer inpatients, particularly among patients undergoing surgery, which can easily lead to pharmacologic overprophylaxis, and thus result in an inherent risk of bleeding (53). Consequently, it is necessary to establish a meticulous benefit and risk assessment model for VTE in patients with malignancies to balance of the risk of bleeding against the risk of thromboembolism. Based on our results, DCA revealed that the nomogram provided superior net benefit of thromboprophylaxis in patients with high VTE risk for threshold probabilities between 2% and 82%. Further large randomised‐controlled trials(RCTs) are needed to evaluate the benefits of thromboprophylaxis in patients at high risk of VTE.

Some limitations of the current study must be considered. First, selection bias could not be avoided due to the single-center retrospective study design. Thus, this model needs to be further validated with larger sample sizes, and/or performed in other centers and other geographic regions to determine its generalizability and efficiency. Second, it is noted that we did not performroutine VTE screening for enrolled patients. This study only identified patients with symptomatic or incidental VTE, which may result in a bias underestimating of the prevalence of VTE in enrolled patients. Moreover, local compression of vascular structures *via* mass lesion and presence of genetic mutations associated with increased thrombosis are risk factors for developing VTEs that cannot be ignored.As a result, limitations of the absence of the above datas must be considered in the current study. Finally, further multi-center prospective clinical trials and community-based prospective studies are needed to validate and refine the model.

## Conclusions

In conclusion, this study systematically developed and validated a novel VTE risk prediction nomogram model for patients with newly diagnosed lung cancer, which incorporated available clinical parameters, genetic and therapeutic factors into the assessment system. Notably, the novel nomogram model was significantly more effective than the existing well-accepted models in routine clinical practice in stratifying the risk of VTE in those patients. We have provided evidence to support that high-performance nomogram model can be reliably used to identify hospitalized patients with lung cancer at a high risk of VTE and to guide individualized decision-making on thromboprophylaxis. However, the model needs external validation in other clinical centers, and should be extended to other care settings (e.g. community-based ambulatory patients).

## Data availability statement

The original contributions presented in the study are included in the article/**Supplementary Material**. Further inquiries can be directed to the corresponding author.

## Ethics statement

Written informed consent was not obtained from the individual(s) for the publication of any potentially identifiable images or data included in this article.

## Author contributions

HL contributed to the study design, statistical analyses, and manuscript writing. YT, HN, LH, GC, CZ and KK performed data collection. HL and YT analyzed and interpreted the data. QL designed and supervised the research and revised the manuscript. All authors read and approved the final manuscript. All authors contributed to the article and approved the submitted version.

## Funding

This work was supported by National Natural Science Foundation of China (grant number 81760014).

## Acknowledgments

The authors are thankful to Mrs. Tiantian Sun and Jiali Lu, biostatistician, for their help and guidance in carrying out the statistical analysis for the study.

## Conflict of interest

The authors declare that the research was conducted in the absence of any commercial or financial relationships that could be construed as a potential conflict of interest.

## Publisher’s note

All claims expressed in this article are solely those of the authors and do not necessarily represent those of their affiliated organizations, or those of the publisher, the editors and the reviewers. Any product that may be evaluated in this article, or claim that may be made by its manufacturer, is not guaranteed or endorsed by the publisher.
