# Supplementary material for: Derivation, validation and assessment of a novel nomogram-based risk assessment model for venous thromboembolism in hospitalized patients with lung cancer: A retrospective case control study
Source: Front Oncol. 2022 Oct 10;12:988287. doi: 10.3389/fonc.2022.988287 (PMC9589115; doi:10.3389/fonc.2022.988287)
Supplement: Supplementary file 1 [file Table_1.docx]

**Supplementary Table 1.** The assessment of risk of bias and applicability for the development and validation of prediction model.

| **Questions** | **Dev** | **Val** | **Rationale** |
| --- | --- | --- | --- |
| **DOMAIN 1: Participants** |  |  |  |
| 1.1 Were appropriate data sources used, e.g. cohort, RCT or nested case-control study data? | Yes/Probably yes | Yes/Probably yes | matched case-control study |
| 1.2 Were all inclusions and exclusions of participants appropriate? | Yes/Probably yes | Yes/Probably yes | Consecutive series of patients included; exclusions appear appropriate. |
| **Overall ROB Domain 1** | **Low risk of bias** | **Low risk of bias** |  |
| **Applicability: Concern that the included participants and setting do not match the review question** | **Low risk of bias** | **Low risk of bias** | The study population of the individual paper matches the targeted population of the review question |
| **DOMAIN 2: Predictors** |  |  |  |
| 2.1 Were predictors defined and assessed in a similar way for all participants? | Yes/Probably yes | Yes/Probably yes | All laboratory examination data were obtained from the pre-treatment baseline assessment after admission. |
| 2.2 Were predictor assessments made without knowledge of outcome data? | No/Probably no | No/Probably no | Retrospective diagnostic model produces bias |
| 2.3 Are all predictors available at the time the model is intended to be used? | No/Probably no | No/Probably no | There are missing values in the state of gene mutation and BMI.Multiple imputation with chained equations was used to replace missing data for BMI values. |
| **Overall ROB Domain 2** | **High risk of bias** | **High risk of bias** |  |
| **Applicability:Concern that the definition, assessment or timing of predictors in the model do not match the review question** | **Low risk of bias** | **Low risk of bias** | Predictors were assessed at presentation and defined in a standard way. They appear to match the review question. |
| **DOMAIN 3: Outcome** |  |  |  |
| 3.1 Was the outcome determined appropriately? | Yes/Probably yes | Yes/Probably yes | Symptomatic or incidental VTE that occurred within the first 6 months of cancer diagnosis during the patients’hospitalization was the primary outcome of the study, including DVT and PE. |
| 3.2 Was a pre-specified or standard outcome definition used? | Yes/Probably yes | Yes/Probably yes | Definition of VTE was provided. |
| 3.3 Were predictors excluded from the outcome definition? | Yes/Probably yes | Yes/Probably yes | DVT and / or PE events confirmed by objective imaging methods |
| 3.4 Was the outcome defined and determined in a similar way for all participants? | Yes/Probably yes | Yes/Probably yes | the outcome of VTE was defined and determined in a similar way for all participants |
| 3.5 Was the outcome determined without knowledge of predictor information? | Yes/Probably yes | Yes/Probably yes | DVT and / or PE events confirmed by objective imaging methods |
| 3.6 Was the time interval between predictor assessment and outcome determination appropriate? | Yes/Probably yes | Yes/Probably yes | The time interval, from enrollment till the end of follow-up was long enough to observe the VTE outcome |
| **Overall ROB Domain 3** | **Low risk of bias** | **Low risk of bias** |  |
| **Applicability:Concern that the outcome, its definition, timing or determination do not match the review question** | **Low risk of bias** | **Low risk of bias** | All items of this domain were explicitly or implicitly addressed. |
| **DOMAIN 4: Analysis** |  |  |  |
| 4.1 Were there a reasonable number of participants with the outcome? | Yes/Probably yes | Yes/Probably yes | The obtained sample size of 234 VTE patients was adequate to address the study aims and a matched sample of 4 non-VTE patients was also obtained. |
| 4.2 Were continuous and categorical predictors handled appropriately? | Yes/Probably yes | Yes/Probably yes | Continuous variables were transformed into categorical variables by determining the optimal cut-off (OCF) value according to the maximum Youden index on the basis of the receiver operating characteristic(ROC) curves. |
| 4.3 Were all enrolled participants included in the analysis? | No/Probably no | No/Probably no | Among the 9819 patients in the control group, only 936 could be matched and included in analyses, whereas the remaining could not be successfully matched were excluded. |
| 4.4 Were participants with missing data handled appropriately? | Yes/Probably yes | Yes/Probably yes | Multiple imputation with chained equations was used to replace missing data for BMI values. |
| 4.5 Was selection of predictors based on univariable analysis avoided? | No/Probably no | - | Variables with a P-value <0.05 in the univariate regression analysis were included in multivariate logistic regression analysis. |
| 4.6 Were complexities in the data (e.g. censoring, competing risks, sampling of controls) accounted for appropriately? | No/Probably no | No/Probably no | This study does not consider the competitive risk to do survival analysis. |
| 4.7 Were relevant model performance measures evaluated appropriately? | Yes/Probably yes | Yes/Probably yes | The model was validated by the consistency index (C-index), receiver operating characteristic curves(ROC) and the calibration plot with the Hosmer-Lemeshow goodness-of-fit test. The clinical utility of the model was assessed through decision curve analysis(DCA). |
| 4.8 Were model overfitting and optimism in model performance accounted for? | Yes/Probably yes | - | The reliability of internal validation was assessed using the bootstrap method with 1000 replicates |
| 4.9 Do predictors and their assigned weights in the final model correspond to the results from multivariable analysis? | Yes/Probably yes | - | The predictors in the final model and their assigned weights correspond to the results of multivariate analysis. |
| **Overall ROB Domain 4** | **High risk of bias** | **High risk of bias** |  |
| **Overall judgement of risk of bias** | **High risk of bias** | **High risk of bias** | Two domains are judged to be at high risk of bias. |
| **Overall judgement of applicability** | **Low risk of bias** | **Low risk of bias** | The evaluation results for all three evaluation areas were low applicability risk |
